# Botulinum toxin type A injections for the management of muscle tightness following total hip arthroplasty: a case series

**DOI:** 10.1186/1749-799X-4-34

**Published:** 2009-08-26

**Authors:** Anil Bhave, Michael G Zywiel, Slif D Ulrich, Mike S McGrath, Thorsten M Seyler, David R Marker, Ronald E Delanois, Michael A Mont

**Affiliations:** 1 Rehabilitation Services, Rubin Institute for Advanced Orthopedics, Sinai Hospital of Baltimore, Baltimore Maryland, USA; 2 Center for Joint Preservation and Replacement, Rubin Institute for Advanced Orthopedics, Sinai Hospital of Baltimore, Baltimore Maryland, USA; 3 Department of Orthopaedic Surgery, Wake Forest University School of Medicine, Winston-Salem North Carolina, USA

## Abstract

**Background:**

Development of hip adductor, tensor fascia lata, and rectus femoris muscle contractures following total hip arthroplasties are quite common, with some patients failing to improve despite treatment with a variety of non-operative modalities. The purpose of the present study was to describe the use of and patient outcomes of botulinum toxin injections as an adjunctive treatment for muscle tightness following total hip arthroplasty.

**Methods:**

Ten patients (14 hips) who had hip adductor, abductor, and/or flexor muscle contractures following total arthroplasty and had been refractory to physical therapeutic efforts were treated with injection of botulinum toxin A. Eight limbs received injections into the adductor muscle, 8 limbs received injections into the tensor fascia lata muscle, and 2 limbs received injection into the rectus femoris muscle, followed by intensive physical therapy for 6 weeks.

**Results:**

At a mean final follow-up of 20 months, all 14 hips had increased range in the affected arc of motion, with a mean improvement of 23 degrees (range, 10 to 45 degrees). Additionally all hips had an improvement in hip scores, with a significant increase in mean score from 74 points (range, 57 to 91 points) prior to injection to a mean of 96 points (range, 93 to 98) at final follow-up. There were no serious treatment-related adverse events.

**Conclusion:**

Botulinum toxin A injections combined with intensive physical therapy may be considered as a potential treatment modality, especially in difficult cases of muscle tightness that are refractory to standard therapy.

## Background

Adductor and tensor fascia lata muscle contractures following total hip arthroplasty are problems that can compromise results. Although certain underlying neuromuscular conditions such as Parkinson's disease may increase the risk of post-operative muscle tightness [1], the reason for contracture development is unclear in many patients. There have been recent attempts using various surgical techniques, including modified and minimally invasive approaches aimed at reducing this complication. In addition, various rehabilitation algorithms have been utilized in combination with these surgical techniques to attempt to further reduce the incidence and severity of these functional contractures [2-4]. Despite the reduction in their overall frequency, persistent contractures remain difficult to treat in some patients and may require further operative intervention. Some of the common surgical treatment modalities include adductor muscle lengthening, tensor fascia lata muscle release, exploration and resection of adhesions, and revision arthroplasty [2,5]. These invasive therapeutic techniques have led to variable outcomes and sometimes only marginal improvements, with reported success in only 0 to 60% of patients [2,6]. These results suggest that secondary surgeries are often not beneficial for patients who fail standard physical therapy interventions, and additional non-operative treatments should be considered.

There is no consensus concerning the best rehabilitation or physical therapy methods to treat patients with severe cases of muscle tightness. After the first indications of stiffness following surgery, mobilization and physical therapy are generally the first steps taken for treatment of muscle contractures. However, there are discrepancies in the reports about the efficacy of various rehabilitation algorithms. When conventional rehabilitation interventions are unsuccessful, more intensive measures such as manual therapy, lidocaine injections, bracing, and manipulation under anesthesia may be employed [2,7]. However, these methods may fail and other non-operative treatment options should be considered.

The use of botulinum neurotoxin has been reported for the treatment of spastic neuromuscular conditions such as cerebral palsy and idiopathic clubfoot [8-11]. The underlying mechanism of action of botulinum toxin occurs at the cellular level, with neuromuscular transmissions blocked as a result of the inhibition of acetylcholine release. By acting selectively on peripheral cholinergic nerve endings, injected botulinum toxin leads to chemodenervation and local paralysis [12]. This temporary paralysis relieves muscle over-activity, which has been shown to be a direct cause of muscle shortening and the associated limitations in both active and passive range of motion [13]. Furthermore, by alleviating muscle over-activity, the botulinum injections also ease extrinsic stretching of the muscle, thus facilitating subsequent therapeutic stretching modalities [14]. The direct clinical effects of temporary paralysis by botulinum toxin in humans have been shown to take affect within a few days and are most effective in the first ten to twelve weeks following treatment [10,15,16]. Based on its ability to block neuromuscular transmissions, and to provide safe and temporary muscle paralysis, botulinum toxin type A was selected as a potential ancillary therapeutic intervention for this study.

The purpose of this study was to describe the use and the patient outcomes of botulinum toxin injections in conjunction with intensive manual joint mobilizations and stretching programs to treat patients with persistent contractures who were recalcitrant to traditional rehabilitation efforts following total hip arthroplasty.

## Methods

The current study encompassed a retrospective review of ten patients (fourteen hips) who received a total hip arthroplasty and had post-operative adductor, tensor fascia lata, and/or rectus femoris muscle contractures. The primary endpoints for this study were an improvement in hip range of motion and functional outcome. The index surgeries were performed between March 2002 and January 2008. The greater majority of total hip arthroplasty patients begin to gain range of motion within 2 to 3 weeks following their index arthroplasty, and continue to improve for up to one year. However, in this study, we specifically addressed a small group of patients who had a markedly different post-operative course. These patients showed a distinct pattern of early post-operative muscle tightness, spasm, and pain, and despite their participating in therapy for up to 2 months following the index arthroplasty, showed no change in range of motion with difficulties participating in rehabilitation efforts. All of these individuals had persistent post-operative muscle contractures, and had no concomitant neuromuscular disease. For this reason, alternative treatments were considered following 2 months of physical therapy. After meeting various exclusion and inclusion criteria, these patients were selected to receive botulinum toxin type A (BoNT/A) treatment, followed by a 6 week physical therapy regimen. Patients were seen daily in physical therapy for 2 weeks following BoNT/A injection, followed by visits 3 times weekly for an additional 4 weeks. Institutional review board approval was obtained for this study. All clinical data collected following botulinum therapy was reviewed and analyzed for this investigation.

### Patient Selection

The inclusion and exclusion criteria used to select total hip arthroplasty patients for botulinum treatment were: A) The patient underwent conventional rehabilitation following their joint replacement surgery; B) The initial rehabilitation outcome was unsuccessful based on having a refractory hip flexion and/or abduction contracture of 10 degrees or more, or an inability to abduct the hip past 10 degrees; C) Adductor and tensor fascia lata muscle contractures were diagnosed based on the individual's complaint of tightness and/or spasms and subsequent clinical evaluation. The clinical tests included assessment of muscle flexibility using the Thomas test for hip flexors [17,18], a modified Ober Test [19] for tightness of the tensor fascia lata and the iliotibial band, and an assessment of hip abduction range of motion for adductor muscle tightness; D) The patient had no existing clinical or radiographic abnormalities such as aseptic or septic loosening, malaligned components, instability, and/or osteolysis. Standard radiographic examinations and clinical evaluations were used to identify individuals with one or more of these exclusion criteria; and E) The patient had no underlying symptomatic spastic neuromuscular disease.

There were two men and eight women who met the established selection criteria. Nine patients had a primary total hip arthroplasty and one patient had contractures following a revision from a bipolar to a total hip arthroplasty. The patients had a mean age of 48 years (range, 19 to 66 years). The underlying diagnosis for the total hip arthroplasty was osteonecrosis in two hips, and osteoarthritis in all remaining cases. Twelve hips were treated with metal-on-metal total hip resurfacing arthroplasty, two hips with stemmed prostheses and a 22 mm head, and the remaining two hips with a stemmed prosthesis and a 26 mm head. After surgery, all patients received standard physical therapeutic modalities at a frequency of 3 times a week. These modalities included range of motion and progressive resisted strengthening exercises and deep heat treatments. The patients showed minimal improvement in both functionality and pain following two or more months of conventional management (except for one at 5 weeks). Common symptoms included: groin pain, back pain, difficulty walking long distances, and limps. Patients also complained of difficulty sitting in, and rising from a chair, as well as descending and ascending stairs. Radiographs revealed well-aligned components with no evidence of loosening and no heterotopic ossification in all patients. Clinical evaluation suggested adductor, rectus femoris, and/or tensor fascia lata muscle rigidity as the primary cause of the patients' symptoms. Based on these findings and the continued poor response to standard rehabilitation techniques, these patients were qualified for inclusion in this study.

### Botulinum Toxin Type A Treatment

**Figure 1:**
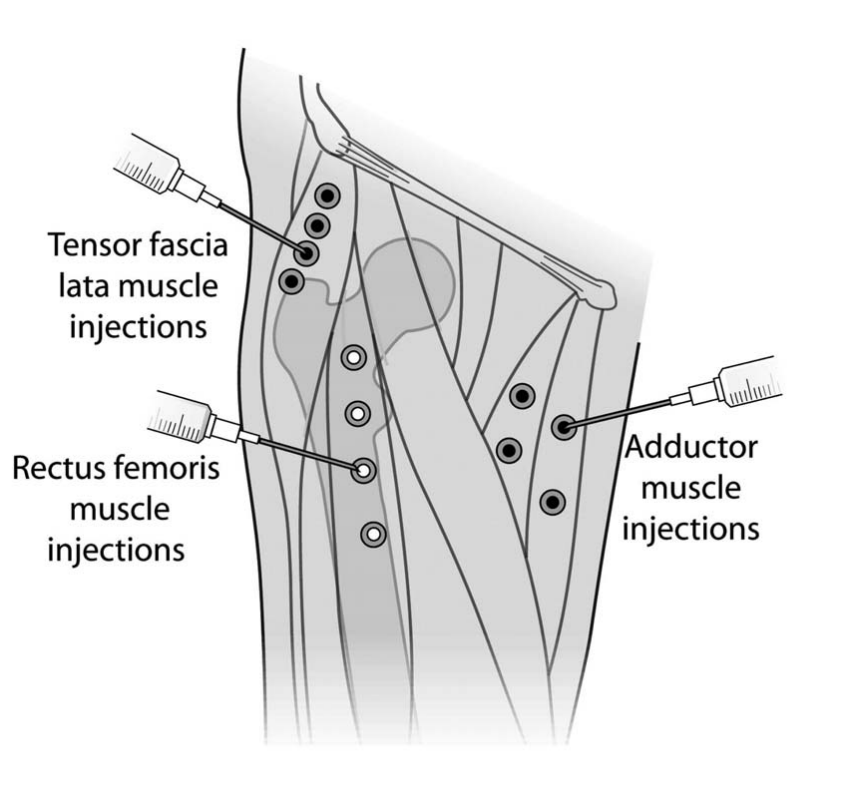
**Botulinum toxin injection points**. Illustration of the locations of the botulinum toxin injections into the adductor, tensor fascia lata, and rectus femoris muscles.

The botulinum toxin type A (BoNT/A) used for treatment in the present study was supplied in vials of 100 units, suspended in 1 milliliter of solution including 0.5 milligrams of human albumin and 0.9 milligrams of sodium chloride (Allergan, Irvine, California). This was further diluted in 4 milliliters of normal saline immediately prior to injection. The mean time from index surgery to treatment with botulinum toxin type A injections was 11 months (range, 1 to 69 months). One patient underwent botulinum toxin treatment five weeks following the index surgery because it was believed that her progress was sufficiently poor that further standard rehabilitation would be of limited benefit. All the remaining patients underwent a minimum of 2 full months of standard rehabilitation following the index arthroplasty. The adductor magnus and brevis muscles were injected in eight limbs, the tensor fascia lata muscle in eight limbs, and the rectus femoris muscle was injected in two limbs. Two patients received injections in both adductor and tensor fasica lata muscles, and two patients received injections in both the tensor fascia lata and rectus femoris muscles. All injections were administered by the senior author (MAM). Patients were placed in a frog leg position for the adductor muscle injection, in a lateral position for the injection of the tensor fascia lata muscle, and in a supine position for injection of the rectus femoris muscle. The injections were performed using a 23 or a 25 gauge needle. The injection sites were identified using a muscle palpation technique, which has previously been described as adequate for the injection of large, superficial muscles [20]. For the adductor magnus and brevis muscles, patients were given dosages of 100 units of botulinum toxin type A at four sites. Similarly, the rectus femoris muscles were injected at four sites with a total of 100 units of BoNT/A, and the tensor fascia lata muscles were injected at four sites with 100 units of BoNT/A (see Figure 1). These dosages were selected based on previously published recommendations for the injection of large muscles [21].

Immediately following the injections, patients were examined for any signs of adverse reaction to the toxin (skin redness, swelling, or systemic symptoms). The patients were also contacted by telephone one and two days following the injections, and evaluated during subsequent clinical visits, to ensure that there were no complications or negative effects due to the toxin. The patients were followed for a median of 17 months (mean 20 months; range, 12 to 62 months) after receiving botulinum therapy.

After receiving the injections, the patients were started on a rehabilitation regime which consisted of outpatient treatment for six continuous weeks. Intensive physical therapy combined with mobilization and stretching of the adductor and abductor muscles was utilized daily for 2 weeks, followed by three times a week for four weeks. All patients received targeted muscle stretching and joint mobilization (Grade III), consisting of holding the joint at the end of their range for 30 seconds, followed by manual application of 15 to 20 oscillations at the end range of motion. All of the manual therapy was performed with opposite limb immobilization and pelvic stabilization. We used modified Ober or Kendall positioning for mobilization of the tensor fascia lata muscle. The rectus femoris muscle was mobilized in the Ely position, and the adductor muscles were stretched in both frog leg and supine positions. Each mobilization was performed for 30 to 45 seconds with end-range oscillations. Typically, patients were treated with 7 to 10 mobilizations for each movement pattern. In addition, all patients received hip flexor mobilization in the Thomas test position. All patients received a standard home exercise program prescribed to all hip arthroplasty patients at our institution for continued joint mobilization and muscle strength maintenance following the completion of formal physical therapy. Only patients with a minimum of 12 months follow-up were included in this report. In the absence of any specific complaints, follow-up intervals after 12 months were on an annual basis as per our institution's standard total hip arthroplasty follow-up guidelines.

Prior to index surgery and throughout follow-up, contracture and arc of motion measurements were made for each subject using a bubble goniometer to assess the clinical outcome. Patients were also evaluated using the Harris hip score rating system [22].

### Statistical Analysis

Data was subjected to averaging and analysis using Sigma Stat software (version 3.00, Systat Corporation, San Jose, California). The clinical outcomes following botulinum toxin type A injection were compared to those prior to treatment using a paired t test, where a p-value of < 0.05 was considered significant.

## Results

All 14 hips achieved a minimum of 10 degrees improvement of arc of motion previously restricted by the contracted muscles, with an overall mean improvement in arc of motion of 23 degrees (range, 10 to 45 degrees).

The eight hips with adductor muscle contractures had a mean improvement in maximum abduction of 31 degrees (range, 20 to 45 degrees), from a pre-injection mean maximal abduction of 8 degrees (range, -5 to 15 degrees) to 38 degrees (range, 15 to 55 degrees) at final follow-up. The eight hips that had tensor fascia lata contractures had a mean improvement in maximum adduction of 16 degrees (range, 10 to 25 degrees), from a pre-injection mean maximal adduction of -9 degrees (range, -15 to -5 degrees) to a mean of 7 degrees (range, -5 to 20 degrees) at final follow-up. Both hips with flexion contractures achieved full extension, with a mean decrease in fixed flexion deformity of 15 degrees (range, 10 to 20 degrees). A comprehensive overview of the results for all treated patients can be found in Additional File 1.

All of the treated hips had Harris hip scores of 80 points or more at the time of final follow-up, and were considered to have a successful clinical outcome. The mean improvement in Harris Hip scores from the time of injection to final follow-up was 22 points (range, 2 to 40 points). The Harris Hip scores improved from a mean of 53 points (range 25 to 75 points) prior to index surgery, to a mean of 74 points (range 57 to 91 points) prior to botulinum toxin injection, and to a mean of 96 points (range 93 to 98 points) at final follow-up (p < 0.001; see Table 1).

**Table 1:** Comparison of mean Harris hip scores prior to surgery, prior to botulinum toxin injection, and at final follow-up

	points	p value
Mean pre-operative Harris hip score	53	
		<0.001
Mean pre-injection Harris hip score	74	
		<0.001
Mean final follow-up Harris hip score	96	

p values were calculated using a paired t-test

There were no serious complications directly associated with the botulinum treatment. Two patients developed mild, transient flu-like symptoms that lasted for two days. However, the patients recovered without further difficulties and showed no noted later effects. Three patients developed redness and swelling at the injection site lasting for several days following treatment, but which resolved within one week in all cases.

## Discussion

There is still controversy concerning the preferred treatment algorithm for muscle contractures following total hip arthroplasty. As previously described, the results of physical therapy have been less than optimal in some difficult cases, and regardless of the specific protocol, there remain a number of cases that do not respond to standard non-operative treatment methods. The present study identified a cohort of patients who continued to show post-operative muscle contractures and unsatisfactory clinical outcomes despite aggressive rehabilitation efforts. This difficult-to-treat patient group was selected to evaluate the effectiveness of using BoNT/A injections as a new ancillary treatment modality in the most severe cases of flexion contracture.

Until recently, there have been few studies indicating that utilized botulinum toxin injections in orthopaedic patients [21,23]. In a study by Alvarez et al., it was used to manage limb spasticity in fifty-one patients with idiopathic clubfoot [8]. None of the patients had prior surgery for clubfoot, and the botulinum toxin was utilized as a substitute for tenotomy to attenuate the function of the triceps surae muscle complex. The patients were followed for a mean of 15 months (range, 1 to 27 months). The results were given for two groups: the first group included patients less than one month old (29 patients), and the second group comprised individuals who were more than one month of age (22 patients). One month following botulinum toxin treatment, the mean ankle dorsiflexion with the knee in flexion improved from 8.0 ± 11.6 degrees to 31.5 ± 11.8 degrees in the first group and 6.1 ± 9.7 degrees to 24.6 ± 9.7 degrees in the second group. The ankle dorsiflexion in flexion remained above 20 degrees for both groups at final follow-up. Similarly, the mean dorsiflexion in extension also improved for both groups, remaining above 15 degrees at a final follow-up of 9 months (range, 1 week to 27 months). Overall, only one patient required limited posterior release and nine patients required repeat manipulation and casting. Based on these results, the study identified botulinum type A as an effective therapeutic approach for idiopathic clubfoot.

To the best of our knowledge, there are only four reports in the literature that evaluated the use of BoNT/A treatment following lower extremity joint arthroplasty. Seyler et al. reported on preliminary results of 11 knee flexion contractures after total knee arthroplasties [24]. Overall, 9 of the 11 knees achieved extension to within 10 degrees of neutral by 2 years following BoNT/A treatment, and 8 of 11 knees maintained this result at a mean follow-up of 36 months. Fish and Chang reported a case of treating iliopsoas tendinitis after a left total hip arthroplasty [25]. The 71 year old woman reported pain that had worsened over a 4 month period and had been made worse by daily activities such as walking and climbing stairs. She showed no signs of periprosthetic infection or malpositioning of the prosthesis. Following injection of 100 units of BoNT/A, both her function and pain showed improvements by 6 months. Her Oswestry Disability index decreased from 26 to 18 points, and her pain intensity numerical rating scale decreased from 7 to 1 on a 10-point scale. The authors suggested that this technique may be an alternative to surgical intervention. Bertoni at al reported the case of a female patient who suffered from complications after total hip arthroplasty [26]. Due to severe pain in the gluteal region not responding to standard treatments, the patient was unable to stand in an upright position or walk, and she was forced to stop her rehabilitation program. Treatment by injection of BoNT/A in the gluteus maximus muscle brought about complete resolution of pain and functional recovery. At final follow up of 16 months, hip assessment confirmed complete pain reduction. Shah et al. reported on the use of botulinum injections for the treatment of a flexion contracture following a total knee arthroplasty in a 61 year old patient with Parkinson's disease [27]. Despite physical therapy, the patient showed little improvement and range of motion was only from 30 to 100 degrees of flexion at one month follow-up. At six weeks after surgery, the patient was injected with 200 units of botulinum toxin type A in the long head of the biceps femoris and the semitendinosus muscle, and at four months he was injected in the gastrocnemius muscle. At final follow-up of 6.5 months, the patient's range of motion improved to 8 to 125 degrees.

Some authors have expressed concerns regarding the cost of using botulinum type A injections to treat muscle spasms [28]. This concern is relevant for patients who suffer from neurological conditions that cause chronic muscle spasms as they would likely require continuous, periodic botulinum treatments. However, none of the patients in this study had flexion contractures associated with a neurological disorder and would ideally require only one treatment. While the authors await long-term results to see if the initial botulinum treatment is sufficient, the mean follow-up of 20 months for this study suggests that a single dose botulinum injection can provide desirable results beyond the ten to twelve weeks in which botulinum is most effective as a neuromuscular transmission inhibitor. No formal physical therapy was required beyond six weeks to maintain these results.

BoNT/A injections may be used with or without local anesthetic, and the toxin has no affect on sensation following treatment. Reports of complications such as fever, pain, local irritation, and redness following BoNT/A botulinum injections are rare, and the effects have only been temporary. While reactions to the injections were not uncommon in our study group, the effects were relatively minor, of short duration, and resolved without any further problems, which was consistent with the low risk level associated with the use of BoNT/A injections.

The limitations of this study include the relatively short-term follow-up, the small numbers of hips (n = 14), and the possible patient selection bias. In addition, this is a retrospective case series with no comparison group, making it impossible to determine whether these patients might have experienced some functional improvement without this treatment method. Although a review of the records did not reveal any sizeable pre-operative muscle contractures, the design of the study did not allow for an accurate assessment of the duration of the pre-operative disability, which might have influenced the post-operative course. Nevertheless, despite these limitations, we did find significant clinical improvement and good outcomes, without severe complications in this difficult to treat group of patients who had not made progress. Although only a preliminary study, we believe that these results provide useful information to the orthopaedic community regarding the treatment of patients with muscle tightness after total hip arthroplasty who are refractory to standard rehabilitation protocols. We are presently studying this treatment modality in a prospective and controlled manner.

## Conclusion

These therapeutic findings suggest that the ancillary use of BoNT/A injections when combined with physical therapeutic modalities improved clinical results for patients with hip adductor and tensor fascia lata muscle tightness who do not respond to conventional treatment modalities. The clinical significance of this study is that patients with contractures who are severely debilitated can have an improved quality of life after BoNT/A, reflected by increased functional abilities and/or decreased pain. Additional studies with larger patient cohorts are currently underway, and the authors await long-term results to see if the promising results of this study can be maintained.

## Competing interests

No external financial support was received in support of this study.

MAM is a consultant for Stryker Orthopaedics and Wright Medical Technologies.

None of the other authors have any financial or non-financial competing interests to disclose.

## Authors' contributions

AB, SDU, TMS, RED, MAM designed the study. MSM, TMS, DRM, RED, MAM collected the data. MGZ, SDU, MSM, DRM analyzed the data. AB, MGZ, SDU, TMS, DRM prepared the manuscript. AB, MGZ, MSM, RED, MAM ensured the accuracy of the data and analysis. All authors have read and approved the final manuscript.

## Supplementary Material

Additional file 1 **Summary of patients treated with BoNT/A injection**. Overview of the demographic profile and pre and post-treatment clinical findings for patients who received botox injection for the management of hip adductor, tensor fascia lata, and/or rectus femoris muscle contractures Click here for file
