# Supplementary material for: Botulinum toxin type A injections for the management of muscle tightness following total hip arthroplasty: a case series
Source: J Orthop Surg Res. 2009 Aug 26;4:34. doi: 10.1186/1749-799X-4-34 (PMC2743655; doi:10.1186/1749-799X-4-34)
Supplement: Additional file 1 — Summary of patients treated with BoNT/A injection. Overview of the demographic profile and pre and post-treatment clinical findings for patients who received botox injection for the management of hip adductor, tensor fascia lata, and/or rectus femoris muscle contractures [file 1749-799X-4-34-S1.doc]

|  | | | | | | | | | | | | | | |  |  |  |  |  |  |  |
| --- | --- | --- | --- | --- | --- | --- | --- | --- | --- | --- | --- | --- | --- | --- | --- | --- | --- | --- | --- | --- | --- |
|  |  |  |  |  |  |  |  |  |  |  |  |  |  |  |  |  |  |  |  |  |  |
|  |  |  |  |  |  | Abduction (degrees) | | | | Adduction (degrees) | | | | Extension (degrees) | | | | Harris Hip Score (points) | | | |
|  | Gender | Age | Side | F/U time (months) | Injection Site | Pre-injection | 6 wks after injection | Final follow-up | Improvement | Pre-injection | 6 wks after injection | Final follow-up | Improvement | Pre-injection | 6 wks after injection | Final follow-up | Improvement | Pre-operative | Pre-injection | Final follow-up | Improvement after injection |
| 1 | F | 31 | L | 18 | Adductor | 5 | 20 | 45 | 40 |  |  |  |  |  |  |  |  | 65 | 91 | 93 | 2 |
|  |  |  | R | 18 | Adductor | 5 | 25 | 45 | 40 |  |  |  |  |  |  |  |  | 65 | 91 | 97 | 6 |
| 2 | F | 47 | L | 19 | Adductor | 10 | 45 | 55 | 45 |  |  |  |  |  |  |  |  | 56 | 86 | 96 | 10 |
|  |  |  | R | 19 | Adductor | 15 | 50 | 55 | 40 |  |  |  |  |  |  |  |  | 56 | 86 | 96 | 10 |
| 3 | M | 66 | L | 17 | Adductor | -5 | 10 | 15 | 20 |  |  |  |  |  |  |  |  | 39 | 57 | 97 | 40 |
|  |  | 66 | R | 17 | Adductor | 10 | 25 | 30 | 20 |  |  |  |  |  |  |  |  | 39 | 57 | 97 | 40 |
| 4 | F | 63 | R | 13 | TFL |  |  |  |  | -10 | 10 | 10 | 20 |  |  |  |  | 61 | 83 | 97 | 14 |
| 5 | F | 57 | R | 19 | TFL |  |  |  |  | -5 | 10 | 20 | 25 |  |  |  |  | 58 | 74 | 98 | 24 |
| 6 | F | 47 | R | 62 | TFL |  |  |  |  | -15 | -5 | -5 | 10 |  |  |  |  | 25 | 65 | 98 | 33 |
| 7 | F | 19 | R | 14 | TFL |  |  |  |  | -10 | 0 | 5 | 15 |  |  |  |  | 40 | 68 | 98 | 30 |
| 8 | M | 48 | L | 12 | Adductor; TFL | 10 | 25 | 30 | 20 | -10 | 0 | 5 | 15 |  |  |  |  | 57 | 61 | 94 | 33 |
|  |  |  | R | 12 | Adductor; TFL | 10 | 25 | 30 | 20 | -10 | 0 | 5 | 15 |  |  |  |  | 60 | 70 | 94 | 24 |
| 9 | F | 38 | R | 17 | TFL; Rectus Femoris |  |  |  |  | -10 | 0 | 0 | 10 | -20 | 0 | 0 | 20 | 43 | 63 | 97 | 34 |
| 10 | F | 55 | L | 24 | TFL; Rectus Femoris |  |  |  |  | -5 | 5 | 15 | 20 | -10 | 5 | 0 | 10 | 75 | 84 | 95 | 11 |
|  | TFL = tensor fascia lata | | | | |  |  |  |  |  |  |  |  |  |  |  |  |  |  |  |  |
